# Incidence and severity of acute mountain sickness and associated symptoms in children trekking on Xue Mountain, Taiwan

**DOI:** 10.1371/journal.pone.0183207

**Published:** 2017-08-23

**Authors:** Fei-Ying Cheng, Mei-Jy Jeng, Yin-Chou Lin, Shih-Hao Wang, Shih-Hao Wu, Wen-Cheng Li, Kuo-Feng Huang, Te-Fa Chiu

**Affiliations:** 1 Institute of Emergency and Critical Care Medicine, School of Medicine, National Yang-Ming University, Taipei, Taiwan; 2 Department of Emergency Medicine, MacKay Memorial Hospital, Taipei, Taiwan; 3 Department of Pediatrics, Taipei Veterans General Hospital, Taipei, Taiwan; 4 Department of Physical Medicine and Rehabilitation, Chang Gung Memorial Hospital at Taoyuan, Taoyuan, Taiwan; 5 Department of Physical Medicine and Rehabilitation, Chang Gung Memorial Hospital at Chiayi, Chiayi, Taiwan; 6 Department of Recreation and Leisure Industry Management, College of Management, National Taiwan Sport University, Taoyuan, Taiwan; 7 Taiwan Wilderness Medical Association, New Taipei City, Taiwan; 8 Department of Emergency Medicine, Dalin Tzu Chi Hospital, Chiayi, Taiwan; 9 Department of Emergency Medicine, Chang Gung Memorial Hospital at Linkou, Taoyuan, Taiwan; 10 School of Medicine, Chang Gung University, Taoyuan, Taiwan; 11 Department of Health Management, Xiamen Chang Gung Hospital, Xiamen, China; 12 Department of Physical Education, National Taitung University, Taitung, Taiwan; 13 Department of Emergency Medicine, West Garden Hospital, Taipei, Taiwan; 14 Department of Emergency Medicine, China Medical University Hospital, Taichung, Taiwan; 15 School of Medicine, China Medical University, Taichung, Taiwan; Armed Forces Medical College, INDIA

## Abstract

**Background:**

Acute mountain sickness (AMS) occurs in non-acclimatized people after an acute ascent to an altitude of 2,500 m or higher. The aim of this study was to examine the incidence and severity of AMS and associated symptoms in children.

**Methods:**

The prospective observational study included 197 healthy, non-acclimatized 11 and 12-year-old children trekking the round-trip from the trailhead to the summit of Xue Mountain, Taiwan (2,179 m to 3,886 m) over 3 days. AMS was evaluated at Qika Hut (2,460 m) on Day 1, at Sanliujiu Hut on Day 2 (3,100 m), and at the same altitude (3,100 m) after reaching the summit on Day 3. We used the Lake Louise Score (LLS) to diagnose AMS and record daily AMS-associated symptoms. We gave acetazolamide to children with mild to moderate AMS. Dexamethasone was reserved for individuals suffering from severe AMS. Acetaminophen was administrated to children with headache, and metoclopramide for those with nausea or vomiting.

**Results:**

There were 197 subjects eligible for analysis. The overall incidence of AMS was 40.6%, which was higher in males and in subjects with a higher body mass index (BMI) (*p* < 0.05). The prevalence of AMS on Day 1 was 5.6%, which was significantly lower than that on Day 2 (29.4%) and Day 3 (23.4%). The mean LLS of all subjects was 1.77 ± 2.08. The overall incidence of severe AMS (LLS ≥ 5) was 12.5%. The mean LLS of the AMS group (3.02 ± 2.46) was significantly higher than that of the non-AMS group (0.92 ± 1.16, *p* < 0.001). Among the AMS group, the mean LLS was 1.00 ± 1.55 on Day 1, 4.09 ± 1.97 on Day 2, and 3.98 ± 2.42 on Day 3. The most common symptom was sleep disturbance followed by dizziness, and headache. The prevalence of headache was 46.2% on Day 2 at 3,100 m, and 31.3% on Day 3 at the same altitude after climbing the summit (3,886 m). Males experienced significantly more headache and fatigue than females (*p* < 0.05). The LLS and prevalence of all AMS symptoms were significantly higher in the AMS than the non-AMS group (*p* < 0.05).

**Conclusions:**

The AMS incidence among children trekking to Xue Mountain was 40.6%. AMS is common and mostly manifests as mild symptoms. Gender (male) and a higher BMI could be considered two independent risk factors of higher AMS incidence. Sleep disturbance is the most common symptom, and the lower prevalence of headache on Day 3 may be due to the effects of medication and/or acclimatization.

## Introduction

Acute mountain sickness (AMS) often occurs in non-acclimatized people after an acute ascent to an altitude of 2,500 m or higher. With the development of modern transportation, more lowland children and adolescents are travelling to high altitudes on school expeditions, or for recreational purposes such as skiing, climbing, and mountaineering. However, previous large group studies on this population are rare. The reported incidence in children varies, and the susceptibility to AMS in this age group compared to adults is controversial [1,2,3]. Several factors contribute to the variations in incidence, including age, AMS definitions, modes and rates of ascent (trekking or by vehicles), and sleeping altitudes [3,4].

The cardinal symptom of AMS is headache, accompanied by other symptoms such as dizziness, lightheadedness, fatigue, weakness, nausea, vomiting, loss of appetite, and sleep disturbance [1,5]. Most AMS symptoms are mild; however, at altitudes above 3,000 m, there is a chance of developing high-altitude cerebral edema (HACE) and high-altitude pulmonary edema (HAPE). In previous reports on adults, HACE usually develops after at least 2 days at altitudes above 4,000 m, and the incidence is estimated to be 0.5–1.0% at 4,000–5,000 m. HAPE develops after 2 or more days at altitudes above 3,000 m, with an incidence of 0.2–2% at 4,500–5,500 m in subjects without a previous history of HAPE [5]. Reports of HAPE and HACE in children are rare, and are usually individual cases. Children who have an upper respiratory infection tend to have a greater chance of developing AMS, and the risk of HAPE, and occasionally HACE, also increases. Children with HAPE have coughs that gradually become worse, and rales on chest auscultation may also be present [1,6].

The diagnostic criteria of AMS were well established in 1991, at the Hypoxia and Mountain Medicine Symposium at Lake Louise, Canada, where a group of experts reached a consensus regarding the diagnosis [7]. The course of AMS is mostly benign and self-limited; it generally occurs within 6–12 hours of ascent [4,5,8,9] and resolves within 1–3 days after descent [5,10]. The major independent risk factors include lack of acclimatization [11,12], rapid ascent, high sleeping altitude [6, 9, 13–15], and a previous history of acute mountain sickness [4,8,15]. However, in one recent study, a history of AMS in children was determined to have no predictive value. None of the children with AMS on re-exposure had AMS the first time, compared with 80% of reproducible AMS among adults [16]. Other factors that may affect the development of AMS are age, gender, residence altitude, concurrent medical illness [1,6,8], and general physical condition [8,9]. Young age is reported to be associated with the development of AMS [8,9,11,15,17–18], while some studies have found no difference between children and adults [2,19,20].

Over the past 30 years, many studies discussed AMS among the adult population. The AMS incidence has ranged from approximately 25% at moderate altitude [8,19,21] to 50–85% at high altitude [17,20,22,23]. However, few studies explored AMS exclusively among children and adolescents, and the results remain inconclusive. Imray et al. reported an incidence of 91.7% in adolescents aged 15–18 years trekking to 4,200–5,500 m in Machu Picchu, Peru [24]. Chan et al. reported an incidence of 59% in older children trekking to Jade Mountain, Taiwan (3,952 m) in 3 days [25]. A group of children and adolescents were studied after rapid ascent from 570 m to 3,450 m in a 3-hour train ride, with a reported incidence of 30–37.5% [3,4]. Theis et al. reported an incidence of 28% in children aged 9–14 years-old at a moderate altitude in Colorado (2,835 m). However, a similar proportion (21%) of children developed these symptoms at sea level. The author cites several important factors other than altitude that may affect the present of symptoms in children, such as travel fatigue, anxiety, and disruption of daily routine [26]. Similar phenomena were also reported in later studies [1].

The aim of this study was to examine the incidence and severity of AMS and associated symptoms in children trekking to Xue Mountain (3,886 m) in Taiwan. AMS was evaluated by trained physicians using the Lake Louise Score (LLS) questionnaire. The prevalence of LLS pertaining to each symptom was also analyzed in order to increase our understanding of AMS in this population.

## Methods

### Study group

This study was performed during a primary school yearly trekking event, and nearly all of the 6^th^ grade students participated in the activity; few dropped out. There were 201 subjects initially; 1 was excluded due to a lack of informed consent, and 3 were excluded due to incomplete data, leaving a total of 197 subjects (age = 11.78 ± 0.43 years; 111 males and 86 females). All subjects were living at altitudes below 500 m. None of the subjects had high altitude exposure (> 2,500 m above sea level) in the previous 3 months. Only two subjects (who took herb medication and *Rhodiola*) had medication before ascent. The protocol was approved by the Institutional Review Board of Chang Gung Memorial Hospital, and written informed consent was obtained from all subjects and their parents.

### Study design

This is a prospective observational study. The subjects, all from the same primary school, were divided into four different trekking groups according to their original classes. The children were separated into four cohorts of 55, 50, 47, and 45 individuals. The departure dates of the groups were separated by 1 day due to limited capacity in mountain huts. The medical team in each group consisted of one emergency physician and one nurse, both of whom were under the leadership of one medical director and one chief nurse for the duration of the trekking activity.

On Day 1, the subjects ascended from Taipei city (5 m above sea level) to the Xue Mountain trailhead (2,170 m) in approximately 6 hours by vehicles. The subjects then trekked 2 km from the trailhead to Qika Hut (2,460 m) in about 2 hours. The evaluation of AMS symptoms and physical examinations were performed by medical staff at Qika Hut in the evening. On Day 2, the subjects trekked for 5 km and ascended to Sanliujiu Hut (3,100 m) in approximately 6 hours. Once again, AMS scoring and physical examinations were performed after adequate rest more than 30 minutes. On Day 3, subjects trekked 3.8 km to the summit of Xue Mountain (3,886 m) in about 5 hours, stayed for less than 1 hour, and descended to Sanliujiu Hut for another AMS evaluation. All participants then descended to the trailhead and were transported home by vehicle. All subjects received the same diet during the trip and care was taken to ensure adequate fluid intake. For subjects who met the criteria of AMS during trekking or at mountain huts, medication (acetaminophen, acetazolamide, metoclopramide, dexamethasone) was provided after evaluation by physicians.

### Evaluation of AMS

The Lake Louise questionnaire was used to evaluate AMS, which included headache, dizziness/lightheadedness, fatigue/weakness, gastrointestinal symptoms (nausea, vomiting, loss of appetite), and sleep disturbance. The sleep disturbance score reflected the sleep quality during the night before evaluation, but the sleep disturbance score of Day 1 was not incorporated in the analysis because all subjects slept at home (near sea level) on the night before trekking. Each symptom was scored on a scale ranging from 0 to 3, where 0 represented the absence of symptoms, 1 indicated mild symptoms, 2 indicated moderate symptoms, and 3 indicated severe and incapacitating symptoms. The sum of all five scores comprised the overall LLS, which ranged from a minimum of 0 to a maximum of 15. Subjects were considered to have AMS if they had a recent altitude gain at an elevation above 2,500 m and had headache, with at least one other symptom plus a total LLS ≥ 3. All evaluations were performed after more than 30 minutes of rest. To decrease subjective bias and the possibility of providing an incorrect answer just “for fun,” serial assessments of AMS symptoms and physical examinations were performed by trained emergency physicians at specific locations, instead of being self-reported by subjects. The medical team consisted of emergency physicians and nurses who had expertise in trekking and high-altitude medicine. The medical staff not only helped the children to complete the LLS questionnaire but also observed the appearance and exercise performance of each child. Objective physical measures, such as resting oxygen saturation (SpO_2_, %) and heart rate (HR, beats per minute), were also obtained.

### Statistical analysis

Continuous data are expressed as means ± SD, and categorical data are presented as percentages. One-way analysis of variance followed by the Student-Newman-Keuls *post hoc* test was used to verify the difference in the LLS on different days. McNemar’s test was used for between-day and within-group comparisons, and chi-squared test was used for between-group comparisons. The Wilcoxon signed rank test was used to detect within-group differences in the score for each symptom, and the two-tailed Mann-Whitney rank sum test was used to compare the score for each symptom between groups. *P*-values < 0.05 were considered statistically significant. All statistical analyses were performed using Sigma Plot software (ver 12; Systat Software, Inc, San Jose, CA, USA).

## Results

The total number of subjects included in the analysis was 197 (age = 11.78 ± 0.43 years; 111 males and 86 females). In total, 80 (40.6%) of the 197 subjects experienced at least one episode of AMS (Table 1): 49 (24.9%) experienced one AMS episode, 29 (14.7%) had two AMS episodes, and only 2 (1.0%) had AMS episodes on three occasions. One subject did not exhibit AMS on either the first or second evaluation; however, the LLS data of the third evaluation were missing. This subject attained the peak and did not receive any treatment during trekking. Thus, the subject was classified into the non-AMS group in terms of overall evaluation. In addition, one subject had a LLS of 10, with all five symptoms presenting on Day 3, which were relieved by medication and immediate descent. None of the subjects developed HAPE or HACE during the trekking course.

**Table 1:** Subject characteristics^d^.

	Total (n^a^ = 197)	AMS group (n^a^ = 80)	Non-AMS group (n^a^ = 117)	*p*-value^d^
Gender				0.033¶
Male, n^a^ (%)	111 (56.3%)	54 (48.6%)	57 (51.4%)	
Female, n^a^ (%)	86 (43.7%)	26 (30.2%)	60 (69.8%)	
Mean body weight, kg	45.1 ± 10.8	47.8 ± 12.1	43.1 ± 9.3	0.008*
Mean body height, cm	152.0 ± 7.1	152.9 ± 7.7	151.3 ± 6.6	0.113
Mean BMI^b^, kg/m^2^	19.4 ± 3.7	20.2 ± 4.0	18.7 ± 3.4	0.008*
Menstruation^c^, n ^a^ (%, of females)	13 (15.1%)	6 (23.1%)	7 (11.7%)	0.398

Abbreviations: AMS, acute mountain sickness; BMI, body mass index. Data are presented as mean ± SD.

^a^ n = number of children

^b^ BMI = weight in kilograms divided by the square of the height in meters.

^c^ In the menstruation section, the total population represents female subjects (86).

^d^ Chi-square test was used to determine differences in categorical variable between the AMS and non-AMS groups. Mann-Whitney U-test was used to determine differences in numerical variables (body weight, body height, BMI) between the two groups.

^¶^ *p* < 0.05 indicates that AMS incidence was significantly higher in males than in females.

* *p* < 0.05 indicates a significant difference between the AMS and non-AMS groups.

Baseline characteristics of all subjects are listed in Table 1. The overall incidence of AMS was significantly greater among males than females (*p* = 0.033): 54 (48.6%) of the 111 males and 26 (30.2%) of the 86 females had AMS. Subjects with AMS had a higher body weight and body mass index (BMI) (*p* = 0.008). Logistic regression, with z-scores for age versus AMS, gender versus AMS, and BMI versus AMS showed that the odds ratio (OR) for age versus AMS was 0.89 (z = -0.75, *p* = 0.455); the OR for gender versus AMS was 1.98 (z = 2.21; *p* = 0.027), and the OR for BMI versus AMS was 1.46 (z = 1.41; *p* = 0.016). Only two subjects had medication before the ascent (herbal medications and *Rhodiola*), and they were both included in the analysis.

**Fig 1:**
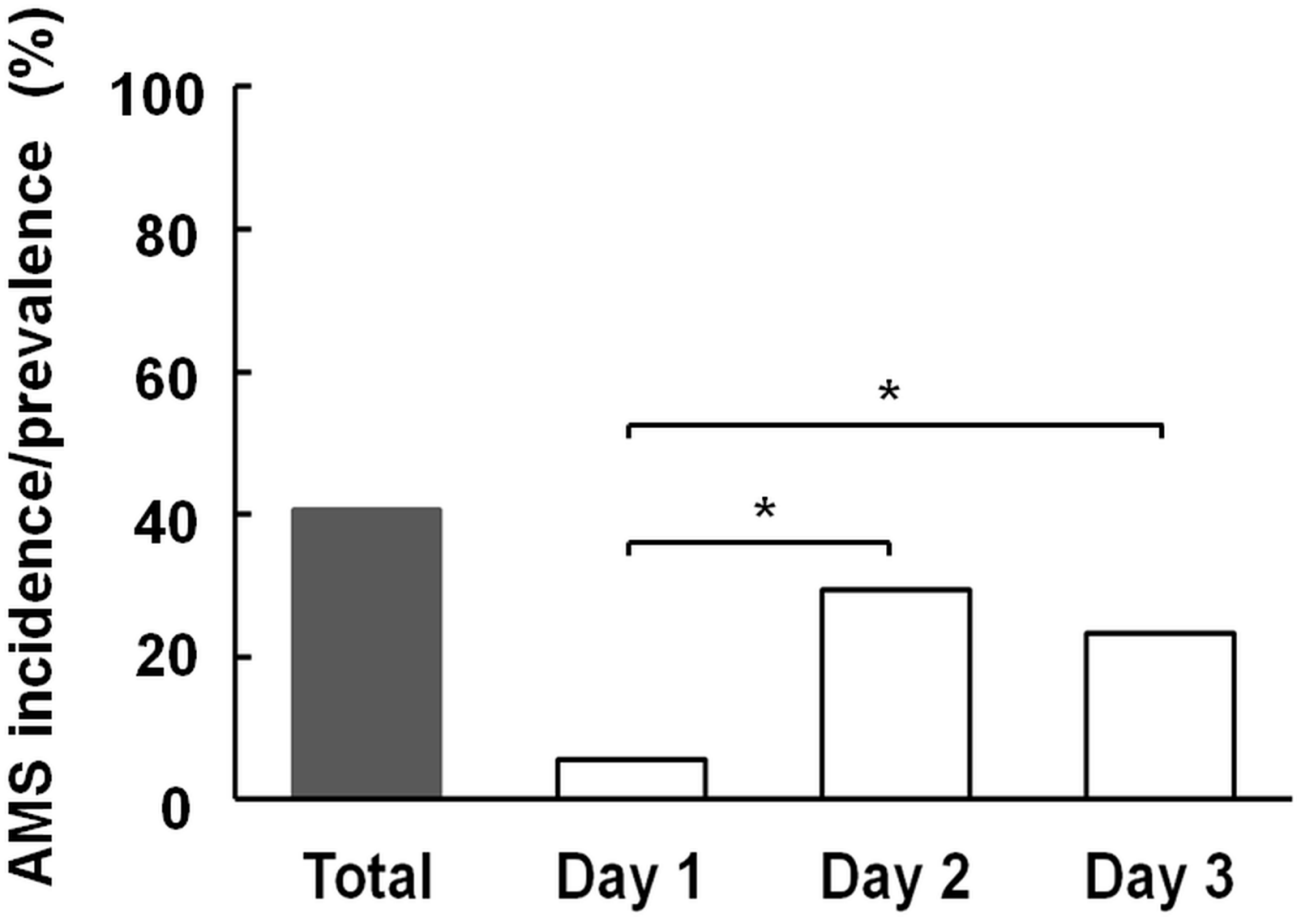
Overall incidence and daily prevalence of AMS in children trekking on Xue Mountain (3,886 m). McNemar’s test was used for between-day comparisons. *P*-values < 0.05 were considered statistically significant. * *p* < 0.001. AMS, acute mountain sickness.

Fig 1 shows that the daily AMS prevalence was significantly higher (29.4%) on Day 2 compared with Day 1 (5.6%; *p* < 0.001). On Day 3, 14 children did not reach Xue Mountain peak: 10 of them had at least one AMS episode during the trip, and 4 did not have AMS. The prevalence of AMS on Day 3 was calculated as follows: 45 (total Day 3 AMS cases) divided by (197–5) (subjects without AMS who failed to reach the peak, and the one subject with missing data) = 23.4%.

For the AMS group, the mean LLS was 1.00 ± 1.55 on Day 1, 4.09 ± 1.97 on Day 2, and 3.98 ± 2.42 on Day 3. The LLS on Day 2 and Day 3 was significantly higher than on Day 1 (*p* < 0.001). The mean LLS of all subjects was 1.77 ± 2.08. In subjects with AMS, the mean LLS (3.02 ± 2.46) was significantly higher than that of the non-AMS group (0.92 ± 1.16; *p* < 0.001). There were three LLS checkpoints for each subject, and 587 (591 minus the 4 subjects without AMS who failed to attain the peak) total occasions were analyzed over the whole trek. Subjects were classified into the AMS group if they experienced AMS at any checkpoint. An LLS of < 2 was observed on 72.5% of all occasions, and an LLS ≥ 5 was found on only 12.5% of all occasions.

A total of 184 participants (93.4%) had at least one of the AMS-associated symptoms. The most prevalent symptom in all subjects was sleep disturbance (74.6%), followed by dizziness, headache, fatigue, and gastrointestinal (GI) symptoms (Table 2). Twenty-six (13.2%) subjects had a total LLS ≥ 3 but fell below the threshold for AMS due to lack of headache. A total of 115 (58.4%) subjects had headache during the trip, but 35 of these headaches (30.4%) did not meet the criteria for AMS. The prevalence of headache was significantly higher (46.2%) on Day 2, and decreased to 31.3% on Day 3 at the same altitude. Sleep disturbance was also most common on Day 2, while fatigue and GI symptoms became more prevalent on Day 3. Over 40% of subjects had dizziness on Day 2 and Day 3.

**Table 2:** Overall prevalence (%) of AMS-associated symptoms on different days^c^.

	Headache	Dizziness	Fatigue	GI symptoms	Sleep disturbance ^b^
**Day 1** ^a^	14.7	14.2	11.2	3.6	N/A
**Day 2** ^a^	46.2*	40.6¶	27.9	12.7	57.9*
**Day 3** ^a^	31.3	41.7¶	32.3*	22.4*	49.5
**Overall**	58.4	61.9	45.7	28.9	74.6

Abbreviations: AMS, acute mountain sickness; GI symptoms, gastrointestinal symptoms.

^a^ Day 1: Qika Hut, 2,460 m. Day 2: Sanliujiu Hut, 3,100 m. Day 3: Sanliujiu Hut, after reaching the summit of Xue Mountain (3,886 m).

^b^ The sleep disturbance score reflected the sleep quality during the night before evaluation, but the sleep disturbance score of Day 1 is not taken into account because all subjects slept at home (near sea level) on the night before trekking.

^c^ McNemar’s test was used to determine the difference in prevalence of each symptom from day to day.

^¶^ *p* < 0.05 indicates that the prevalence of dizziness was higher on Day 2 and Day 3 compared with Day 1. However, there was no significant difference between Day 2 and Day 3.

* *p* < 0.05 indicates a statistically higher prevalence compared with other days.

**Fig 2:**
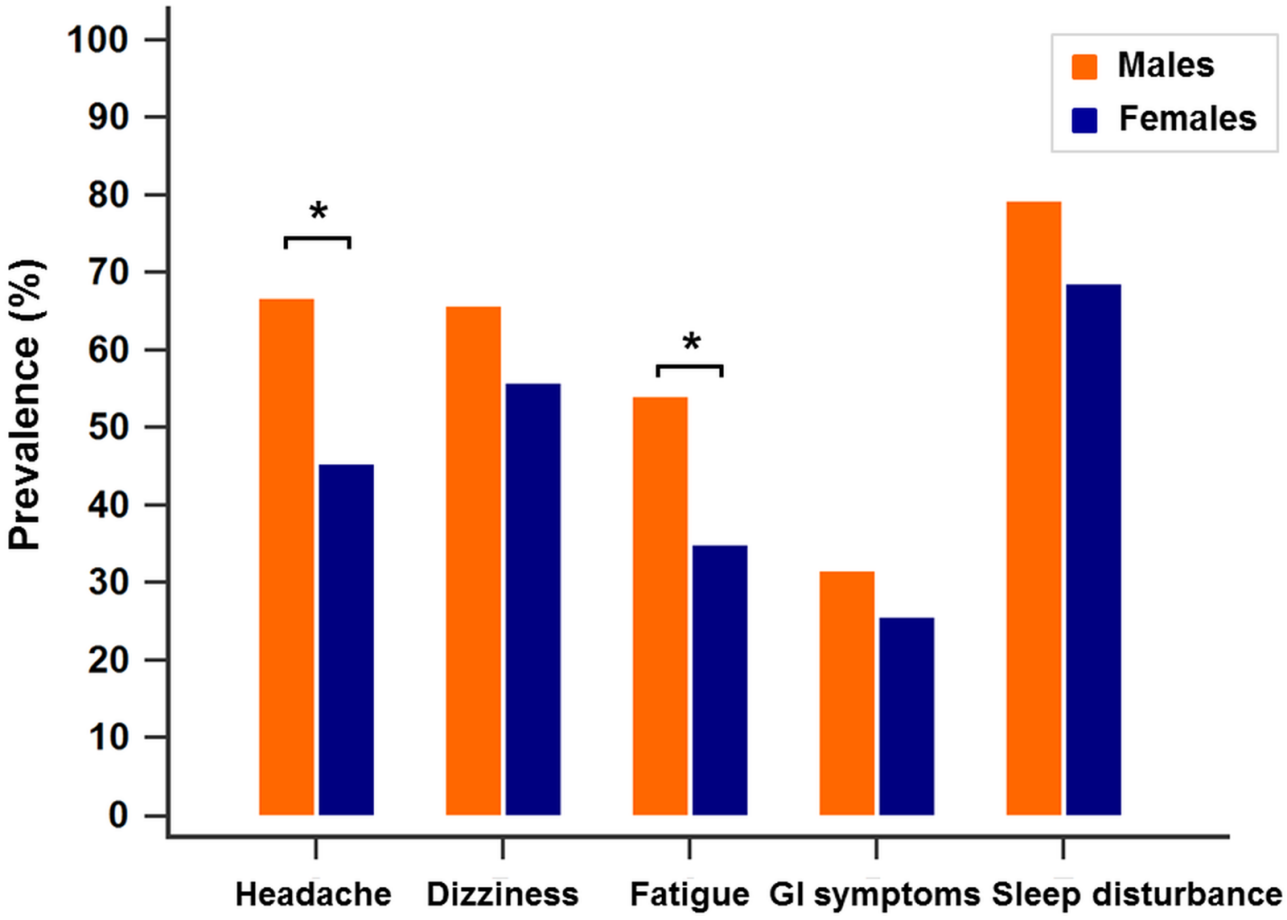
Prevalence of AMS-associated symptoms in males and females. The prevalence of headache and fatigue was significantly higher among males than females. More than half (67.7%) of the male subjects experienced headache compared to 45.4% of females. The prevalence of fatigue was also higher among males (54%) than females (34.9%). The chi-squared test was used to detect differences in prevalence between male and female subjects. *P*-values < 0.05 were considered statistically significant. * *p* < 0.05. AMS, acute mountain sickness; GI symptoms, gastrointestinal symptoms (nausea, vomiting, poor appetite).

Fig 2 shows the prevalence of AMS-associated symptoms in males and females. More than half (67.7%) of the male subjects experienced headache compared with 45.4% of female subjects (*p* < 0.05). The prevalence of fatigue was also higher among males (54.0%) than females (34.9%) (*p* < 0.05).

**Fig 3:**
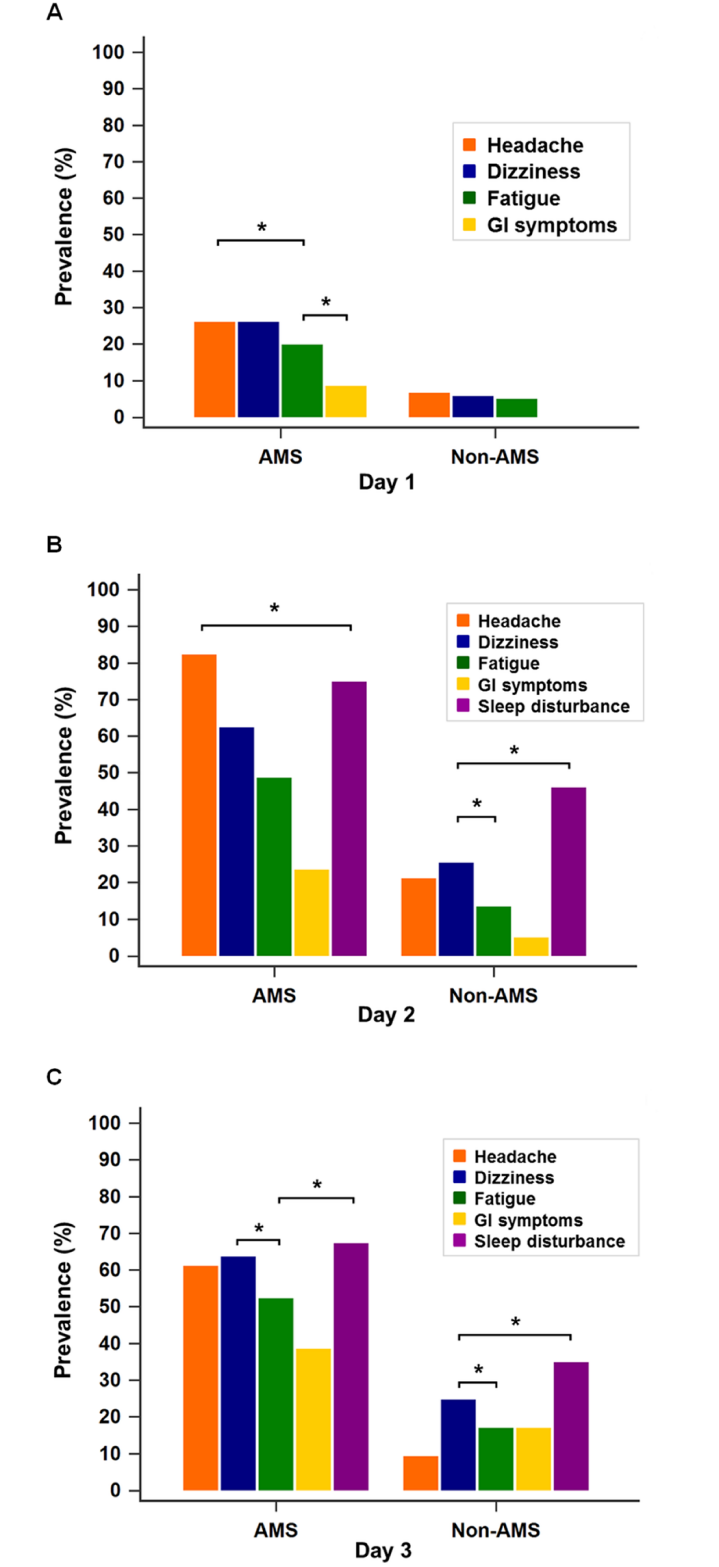
Daily prevalence of AMS-associated symptoms in the AMS and non-AMS groups. A—Day 1 (Qika Hut, 2,460 m): 26% of the subjects in the AMS group experienced headache and dizziness. Fewer than 7% of the non-AMS group experienced AMS symptoms, and none experienced GI symptoms at this altitude. B—Day 2 (Sanliujiu Hut, 3,100 m): headache was the most prevalent symptom (82.5%) in the AMS group, followed by sleep disturbance. In the non-AMS group, the main symptom was sleep disturbance (46%) followed by dizziness, headache, and fatigue (prevalence < 30% for all). C—Day 3 (Sanliujiu Hut, 3,100 m, after reaching the summit of Xue Mountain, 3,886 m): more than 50% of the AMS group experienced headache, dizziness, fatigue, and sleep disturbances. Sleep disturbance was the most prevalent symptom in both groups. All AMS symptoms were significantly more prevalent in the AMS than the non-AMS group on all 3 days. The sleep disturbance score reflected the sleep quality during the night before evaluation, but the sleep disturbance score on Day 1 was not considered in the analysis because all subjects slept at home (near sea level) on the night before trekking. McNemar’s test was used for within-group comparisons of prevalence, and the chi-squared test was used for between-group comparisons. *P*-values < 0.05 were considered statistically significant. * *p* < 0.05. AMS, acute mountain sickness; GI symptoms, gastrointestinal symptoms (nausea, vomiting, poor appetite).

**Fig 4:**
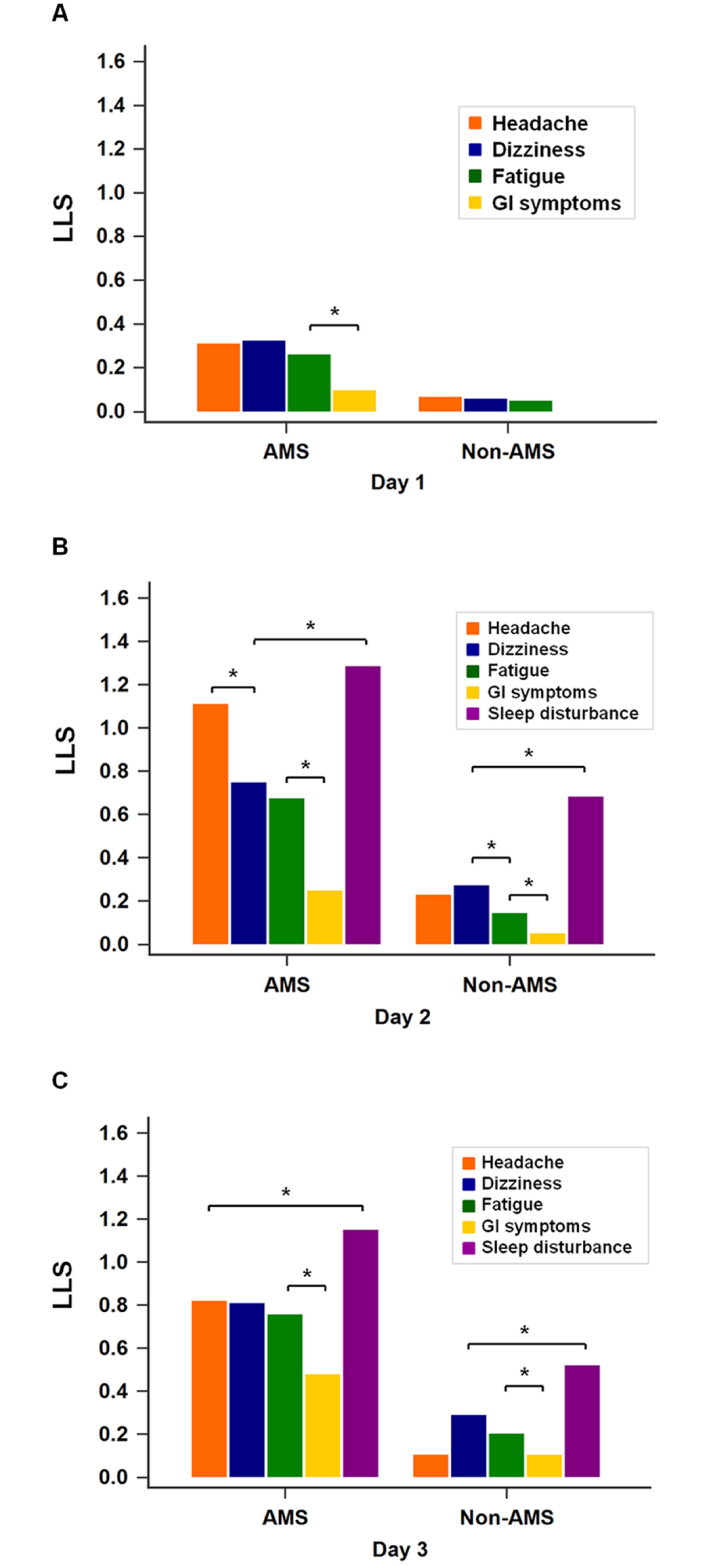
LLS for each symptom according to group on days 1–3. A—Day 1 (Qika Hut, 2,460 m): the gastrointestinal (GI) symptom score was lowest in the AMS group. No subject in the non-AMS group experienced GI symptoms at this altitude. B—Day 2 (Sanliujiu Hut, 3,100 m): the sleep disturbance score was the highest LLS in both groups (AMS: 1.29 ± 0.92, non-AMS: 0.68 ± 0.83). In the AMS group, headache and dizziness were the most severe symptoms after sleep disturbance. C—Day 3 (Sanliujiu Hut, 3,100m, after reaching the Xue Mountain summit, 3,886 m): similar to previous days, the sleep disturbance symptoms were the most severe, and GI symptoms the least severe, in both groups. On all 3 days, all of the LLS were significantly higher for the AMS group than for the non-AMS group. The sleep disturbance score reflected the sleep quality during the night before evaluation, but the sleep disturbance score of Day 1 was not considered in the analysis because all subjects slept at home (near sea level) on the night before trekking. The Wilcoxon signed rank test was used to detect within-group differences in the LLS for each symptom, and the Mann-Whitney rank sum test was used to compare LLS for each symptom between groups. *P*-values < 0.05 were considered statistically significant. * *p* < 0.05. LLS, Lake Louise Score; AMS, acute mountain sickness; GI symptoms, gastrointestinal symptoms (nausea, vomiting, poor appetite).

The daily prevalence and score for each symptom are shown in Figs 3 and 4. On Day 1, the main symptoms in both groups were headache and dizziness, with only a few GI symptoms reported (Fig 3A). Fewer than 7% of the non-AMS group experienced AMS symptoms, and the LLS for each symptom in both groups were all less than 0.4 on Day 1 (Fig 4A). On Day 2, headache was the most prevalent symptom (82.5%) among the AMS group, followed by sleep disturbance and dizziness, while in the non-AMS group, the main symptom was sleep disturbance (46%) followed by dizziness, headache, and fatigue (prevalence < 30% for all) (Fig 3B). Sleep disturbance had the highest LLS in both groups (AMS: 1.29 ± 0.92, non-AMS: 0.68 ± 0.83) on Day 2 (Fig 4B). On Day 3, more than 50% of the AMS group experienced headache, dizziness, fatigue, and sleep disturbance (Fig 3C). Sleep disturbance symptoms remained the most prevalent and severe, while GI symptoms the least severe in both groups (Fig 4C). On all three days, the LLS and prevalence of all symptoms were significantly higher in the AMS versus non-AMS group (*p* < 0.05) (Fig 4A–4C).

On Day 1, the HR was 113.69 ± 11.51 in the AMS group and 112.37 ± 12.22 in the non-AMS group, while the SpO_2_ (%) was 91.28 ± 2.24 in the AMS group and 91.42 ± 2.40% in the non-AMS group. On Day 2, the HR was 114.70 ± 11.21 in the AMS group and 111.38 ± 11.92 in the non-AMS group, while the SpO_2_ was 87.59 ± 3.88% in the AMS group and 87.99 ± 4.50% in the non-AMS group. On Day 3, the HR was 119.65 ± 14.85 in the AMS group and 117.82 ± 11.90 in the non-AMS group, while the SpO_2_ was 87.49 ± 6.31% in the AMS group and 87.73 ± 4.62% in the non-AMS group. There were no significant differences in HR and SpO_2_ between the groups on any day.

A total of 73 (37.1%) children received medication during the trip. Among the AMS group (n = 80), 45 (56.3%) received medication. Of the non-AMS group (n = 117), 28 (23.9%) were given either acetaminophen for headache/dysmenorrhea, or metoclopramide for GI symptoms such as nausea/vomiting. Notably, we gave acetazolamide and/or dexamethasone only to children who met the criteria for AMS (headache plus an LLS ≥ 3).

## Discussion

In Taiwan, the number of trekkers going to Xue Mountain has increased steadily over the past two decades, to more than 25,000 per year. The increase in popularity is associated with the convenience of reaching the trailhead by vehicle, good trail conditions for most of the year, and the subtropical climate of Taiwan. The number of children and adolescents going to high altitudes also increased, and it has become more important to have a better understanding on the incidence, risk factors, and common symptoms of AMS among this population. The AMS incidence increased with altitude [10,22,24,27]. In a study led by Imray et al., 91.7% of adolescents had AMS while trekking to 4,200–5,500 m [24]. Major et al. reported a similar incidence (84%) in a group of adolescents ascending to 5,200 m during a 23-day expedition [23]. In previous studies, it was unclear whether young age was a risk factor of AMS [8,9,11,15,17–18]. Gonggalanzi et al. found an AMS incidence of 36.7% among tourists (mean age = 37.2 ± 14.4 years) visiting the city of Lhasa, which is at 3,658 m [9]. Moraga et al. reported an AMS incidence of 27% in 15 tourists with a mean age of 31.9 years and 50% in 10 tourists with a mean age of 15.4 years and ascending to 3,500 m by truck [18]. In a study conducted at the Swiss high altitude research station at Jungfrau (3,450 m), AMS prevalence was lower in children than in adolescents and adults on Day 1 (8–10 hours within ascent), but not on Day 2. The cumulative prevalence of AMS was 30%, 37%, and 45% in children, adolescents, and adults, respectively (*p* < 0.001) [3]. In our investigation, the overall incidence of AMS among children aged 11–12 years and trekking to Xue Mountain (3,886 m) was 40.6% (Table 1). Our results are comparable to those of several previous studies of children travelling to similar altitudes [4,14]. In addition, the trekking route, sleeping altitude, and the 3-day ascent profile of Xue Mountain is similar to that of the Jade Mountain (3,952 m) trail in Taiwan. The AMS incidence in children was higher compared with adult climbers on Jade Mountain, which was reported to be 32–36% by Wang et al. and Chen et al. [11,15]. Although AMS was common among children, our study revealed that the AMS symptoms were mostly mild. For all occasions, 72.2% of subjects had LLSs < 2. None of the subjects in our study developed HAPE or HACE during the trekking course.

The rate of ascent and sleeping altitude also have an important impact on the incidence of AMS [1,5,6,8,13–15]. However, the sleeping altitudes differed among previous studies, as did the ascent rate and method (by foot, vehicle, or airplane [4,9,14,21]). There were also other confounding factors, such as age, gender, physical ability, and concurrent illness [1,6,8]. Therefore, it was difficult to compare these studies [3]. The purpose of our study was to evaluate the incidence and severity of AMS among children for this particular trekking route.

Although the prevalence and severity of AMS increases with a recent gain in altitude, there was evidence that subjects experienced less AMS and AMS-associated symptoms after a previous stay at high altitudes [15,20,24,28]. In teenagers travelling between 2,400 m and 5,500 m, the LLS decreased despite the subjects ascending to over 5,000 m on the 13^th^ to 18^th^ days of the expedition [24]. Wang et al. found that the most common site of AMS symptom development on Jade Mountain was the midway overnight hut, the Paiyun Lodge (3,400 m), but not on the summit or on the day after reaching the summit [15]. In our study, the prevalence of AMS increased from 5.6% on Day 1 to 29.4% on Day 2, and slightly decreased (23.4%) despite the effort to reach the summit of Xue Mountain (3,886 m) on Day 3 (Fig 1). There was also a trend of decreasing AMS severity at the same altitude (the mean LLS of AMS group was 4.09 ± 1.97 on Day 2, and 3.98 ± 2.42 on Day 3). The decrease in AMS prevalence and severity was mostly due to the decrease in headache prevalence and severity (Figs 3B, 3C and 4B and 4C), which was most common (46.2%) on Day 2 and decreased significantly to 31.3% on Day 3 (Table 2). Although there was a decreasing trend in AMS prevalence and severity on Day 3, the comparison between Day 2 and Day 3 revealed no statistical difference. The effects of medication and/or acclimatization may have contributed to this finding.

Most studies found no difference in AMS incidence between males and females [4,14–15,21,25,29], while some studies reported that female sex was more susceptible to the development of AMS [8,13,20,24,27–28]. However, our study showed that males had a significantly higher incidence of AMS than females (Table 1). There was also a trend toward males’ having a higher overall prevalence of AMS-associated symptoms, with headache and fatigue being significantly more prevalent than in females (Fig 2). Higher AMS incidence was also seen in subjects with a higher BMI. Gender (male) and BMI can be considered as two independent risk factors for AMS. The pubertal stage of each child was not evaluated, since this was not part of our study protocol. Most of the girls had reached puberty (and almost all of them had periods), while half of the boys had not experienced a voice change at the time of the activity. Hormones may play a role in acclimatization to high altitude, but the underlying mechanism remains unknown and requires further investigation.

A total of 73 (37.1%) children received medication during the whole trekking course. Among the AMS group (n = 80), 45 (56.3%) received medication. Of the non-AMS group (n = 117), 28 (23.9%) received medication (either acetaminophen for headache/dysmenorrhea or metoclopramide for nausea/vomiting). We gave acetazolamide and/or dexamethasone only to children who met the criteria for AMS (headache and an LLS ≥ 3) so that the administration of acetazolamide and/or dexamethasone would not affect the overall incidence of AMS. The use of acetaminophen for treating headache may have been a confounding factor, since headache is required for an AMS diagnosis. Less than 30% of the children experienced GI symptoms, which were also the least prevalent AMS-associated symptom. The administration of metoclopramide may also have affected the result; however, the effect was minimal. In addition, we studied an annual trekking activity of primary school children. To ensure the safety of the children, we administered treatments immediately if needed. Due to ethical concerns, we could not permit the possibility of the children experiencing severe AMS for research purposes. Administering medication may lead to underestimating of the overall incidence of AMS, as well as the daily prevalence and severity of AMS. Of note, one subject had taken herbal medicine before activity, and one took *Rhodiola* as a prophylaxis to AMS. In a recent randomized double-blind crossover trial, *Rhodiola* extract was ineffective in reducing the incidence or severity of AMS compared with a placebo [30].

Headache was the most prevalent AMS-associated symptom in most previous studies [8,15,17,24]. On the other hand, Bloch et al. and Gonggalanzi et al. reported fatigue to be the most common symptom at around 3,500 m [4,9]. In our study, 115 (58.4%) subjects experienced at least one episode of headache during the trip (Table 2). Furthermore, 35 of these did not have other symptoms or an LLS ≥ 3. Twenty-six (13.2%) subjects had an LLS ≥ 3 but did not meet the criteria for AMS. These may indicate early or mild AMS. The most common symptom of AMS in our study was sleep disturbance, followed by dizziness, headache, fatigue, and GI symptoms (Table 2). We found that up to 74.6% of children had sleep disturbance during the trip (Table 2), which was a higher rate than in other studies [8,22,24]. Similar findings were also reported in children and adult trekkers on Jade Mountain (3,952 m) [15,25]. This may be due to the crowded environment of Qika Hut and Sanliujiu Hut, which contain a maximum of 130 and 110 people, respectively, in two large rooms. Different mountain trekking groups have different times of arrival and departure, and the noise may disturb the sleep of other teams. A similar situation was observed by Horiuchi et al. on Mount Fuji (3,776 m), where climbers staying overnight at a lodge were more likely to have poor sleep quality and a reduction in total sleep time [29]. Moreover, it was likely that cold weather, and not being accustomed to sleeping bags, contributed to the higher sleep disturbance scores. Several children also reported that they missed their homes and parents at night, which made it difficult to fall asleep. The other AMS-associated symptoms may be the result of travel fatigue and physical effort expended while trekking. Children traveled in vehicles for 6 hours, and trekked for 2 hours on Day 1; thus, they were likely to have symptoms, such as fatigue or nausea, due to travel that might have imitated the milder forms of AMS. These special considerations, together with other factors, such as boredom and disruption of daily routine, among this population have also been reported in previous literature [1,26].

Finally, medical teams were on standby and trekked with each group. We also established a “high-altitude clinic” at Sanliujiu Hut (3,100 m), where a senior emergency physician and an experienced nurse were stationed to deliver medical services at night during the trek. Once a subject developed AMS or other unbearable symptoms, the physicians would evaluate and treat them immediately. Early detection of AMS might have prevented the children from experiencing serious complications. This was the first established medical service provided for children at high altitudes in Taiwan. More than 2,000 children have benefited from the medical service since 2009 [25,31]. Although we measured HR and SpO_2_, there were no significant differences in either measure between the AMS and non-AMS group on any day. We did not discuss this in this article, as the effect of acetazolamide may have influenced these physiologic measures.

### Study limitations

The major limitation of this study was the medication administrated during the trip. This may have led to underestimating of the overall incidence of AMS. For ensuring the children’s safety, we treated subjects with AMS and other complaints. We administrated acetaminophen for headache, metoclopramide for nausea/vomiting, acetazolamide only when subjects met the criteria for AMS, and dexamethasone for severe AMS. The medication was given at any place or time along the trekking route once a child developed AMS or other symptoms. Since the medication treatment plan was not part of our study protocol, we did not analyze the time of treatment or any subsequent effects of medication. Thus, the daily AMS prevalence and severity were also possibly subject to bias and underestimation. Second, four subjects were unwilling to ascend to the summit or (because of other discomfort that did not meet the criteria for AMS) descended to Qika Hut. These four non-AMS subjects were excluded from the analysis of AMS prevalence and LLS on Day 3, which may also have affected the results. Third, the mean BMI of the males (19.79 ± 4.29 kg/m^2^) was slightly higher than that of the females (18.83 ± 2.75 kg/m^2^); however, there was no significant statistical difference (*p* = 0.453). Although we have adjusted the associations among gender, BMI, and AMS using logistic regression and z-scores, this may still have affected the results regarding the association between BMI and AMS prevalence. Finally, it is possible that some confounding factors, such as poor sleep conditions, changes in routine and diet lifestyle, and absence of family members may have accounted for the symptoms such as sleep disturbance.

## Conclusions

The AMS incidence among children trekking to Xue Mountain was 40.6%. Gender (male) and a higher BMI can be considered as two independent risk factors for AMS. Although AMS was common among children, the symptoms were mostly mild. Sleep disturbance was the most common symptom. The effects of medication and/or acclimatization may explain the lower incidence of AMS and headache on Day 3 compared to Day 2.
